# Meteorological Register for February, 1841

**Published:** 1841-03-06

**Authors:** J. Conrad


### METEOROLOGICAL REGISTER FOR FEBRUARY, 1841.

*Kept at the Pennsylvania Hospital, by J. Conrad.*

| Date. | Thermometer. | | | Barometer. | | Dew Point | Winds. | | Rain. | REMARKS. |
|---|---|---|---|---|---|---|---|---|---|---|
| | Max. | Min. | 9 AM. | 10 AM | 3 PM. | | Direction. | Force | | |
| 1 | 37 | 32 | 36 | 29.90 | 29.57 | 31 | NW.NE. | 2 | .780 | Sleet from 8½ AM. |
| 2 | 38 | 31 | 33 | 29.95 | 29.91 | 27 | NW.E. | 0 | | Cloudy. |
| 3 | 41 | 27 | 38 | 29.80 | 29.97 | 22 | SW.NW. | 2 | .005 | Clear; light showers in mor'g. |
| 4 | 36 | 23 | 30 | 30.37 | 30.31 | 8 | NW. | 1 | | Clear. |
| 5 | 39 | 23 | 26 | 30.21 | 30.07 | 21 | NE. | 1 | | Clear. |
| 6 | 44 | 28 | 35 | 30.01 | 30.01 | 30 | NE.SW. | 1 | | Cloudy. |
| 7 | 38 | 32 | 37 | 30.21 | 30.20 | 24 | N. | 1 | .007 | Cloudy; snow from 11 AM. till 4 PM. |
| 8 | 37 | 27 | 30 | 30.38 | 30.31 | 18 | N. | 1 | | Clear. |
| 9 | 36 | 27 | 33 | 30.09 | 29.94 | 30 | NE. | 1 | .360 | Snow from 11 AM. all day. |
| 10 | 38 | 16 | 29 | 29.94 | 29.92 | 9 | SW.NW. | 2 | | Clear; snow squall at 1½ PM. |
| 11 | 20 | 8 | 14 | 30.10 | 30.00 | —6 | NW. | 2 | | Clear. |
| 12 | 15 | 3 | 8 | 30.08 | 30.00 | —7 | W.NW. | 2 | | Clear. |
| 13 | 20 | 6 | 13 | 29.98 | 29.97 | —2 | W.NW. | 2 | | Clear. |
| 14 | 28 | 13 | 20 | 29.97 | 29.93 | 4 | N.NW. | 2 | | Morning cloudy; afternoon clear. |
| 15 | 25 | 14 | 17 | 30.02 | 30.02 | 4 | NW. | 3 | | Clear. |
| 16 | 36 | 14 | 26 | 30.08 | 29.98 | 16 | SW. | 2 | | Clear. |
| 17 | 41 | 28 | 34 | 29.77 | 29.82 | 24 | SW.NE. | 1 | | Morning partly clear; afternoon cloudy. |
| 18 | 33 | 26 | 28 | 30.15 | 30.08 | 18 | NE.SE. | 1 | | Morning cloudy; afternoon clear. |
| 19 | 42 | 26 | 34 | 29.70 | 29.67 | 24 | SW.NW. | 3 | | Cloudy; evening clear. |
| 20 | 44 | 25 | 30 | 29.89 | 29.79 | 17 | NW.SW. | 2 | | Clear. |
| 21 | 51 | 26 | 35 | 29.65 | 29.59 | 25 | SW. | 0 | | Morning clear; aftern'n cloudy. |
| 22 | 47 | 34 | 38 | 29.73 | 29.68 | 31 | NE. | 0 | | Clear. |
| 23 | 58 | 27 | 40 | 29.48 | 29.63 | 23 | SW.N. | 3 | | Clear; evening cloudy. |
| 24 | 26 | 20 | 22 | 30.21 | 30.17 | 15 | NE. | 1 | .047 | Snow from 7½ AM. till 2½ PM; evening clear. |
| 25 | 34 | 13 | 21 | 30.29 | 30.24 | 23 | SW. | 1 | | Morning clear; afternoon cloudy. |
| 26 | 48 | 32 | 38 | 30.17 | 30.09 | 32 | NW.SE. | 0 | | Clear; evening cloudy. |
| 27 | 47 | 35 | 44 | 29.74 | 29.78 | 36 | SW.NW. | 1 | .188 | Rain in morning; evening clear. |
| 28 | 52 | 32 | 40 | 30.07 | 30.01 | 32 | SW. | 2 | | Clear. |
| Mean | 37.53 | 23.14 | 29.60 | 29.99 | 29.95 | 18.76 | | | 1.387 | |

| | | | |
|---|---|---|---|
| Mean temperature, | 30.33° | Clear days, - - | 17 |
| " pressure, | 29.981 inches. | Clouds, rain, &c. - | 11 |
| " dew-point, | 18.76° | | |

*Winds*—S. to W. 9 days; W. to N. 9 days; N. to E. 8½ days; E. to S. 1½ days.

